# 

**Published:** 1897-01

**Authors:** 


### INDEX TO VOLUME LI.

PAGE.

COMMUNICATIONS.

Abrasions of the Teeth......... 157
Action of Arsenious Acid upon the Tooth-Pulp......... 105
Address........................... 261
Address by Chairman............ 377
Address by President........... 417
Anterior Pillars of the Fauces —Etiology and Treatment 391
An Obscure Case.................. 574
A Suggestion or Two............ 275
Bacteriology—Dental............ 441
Bridge Work....................... 560
Cataphoresis vs. Direct Application of the Galvanic Current for Obtunding Sensitive Dentine; and How to Conduct a Practice that Excludes Both... 381
Cataphoresis — The Reason Why We Fail............... 66
Cataphoresis and Anaphoresis 224
Continued Study of Relations of the Frontal Sinus to the Antrum .................. 562
Defense of Theory............... 62
Dental Legislation............... 53
Dental Practice.................... 490
Distribution of Exostosis of the Teeth....................... 174
Education of the People About the Teeth....................... 217

ELECTRICITY IN DENTISTRY—

Electricity in Dentistry...... 220
Electrolysis in Dentistry..... 221
Experience in Dentistry........ 265
Facial Line and Angles in Prosthetic Dentistry........ 556
Frontal Sinus Communicating with the Antrum of Highmore.......................... 573
Galvanic Current................ 92
Glance at Some Relations of Dentistry to General Medicine............................ 389

PAGE.

How to Conduct a Dental Practice in an Agricultural District................ 501
How to Conduct a City Practice ........................... 507
Hygiene of the Teeth........... 313
Hyperæmia and Inflammation of the Dental Pulp......... 395
Implantation of Sterilized Roots of Animals' Teeth Carrying Artificial Crowns 394
Judgment.......................... 278
Libby Method..................... 563
Manifestations of Abrasion..... 176
Misapplication of Gold Crowns in Bridgework............. 561
Need of Dental Instruction in Medical Schools............. 292
Nodule on Apex of Root....... 168
Opening the Bite with Cap-Fillings......................... 576
Orbicularis Oris, and the Muscles of Expression.......... 1
Orthopædia......................... 550
Pathologic Conditions of the Pharynx and Contiguous Structures During Early Childhood — Prime Factors in the Etiology of Mal-Formed Maxillæ and Irregular Teeth, etc........ 387
Photo-Micography ............... 564
Phonomena of Cataphoresis... 525
Point of Contact.................. 425
Porcelain Inlays.................. 270
Pyrozone—Its Uses............... 281
Relative Efficiency of Various Current-Controllers for Cataphoresis................... 76
Relation of Chemistry and Bacterology of the Stomach to the Dental Caries.. 469
Relation of Chemistry to Dentistry.......................... 546
Replantation in Pyorrhea Alveolaris.......................... 569

PAGE.
Resection and Reproduction of the Maxillæ.................. 373
Report of Committee on Nomenclature.......... ..... 169
Section II.—Report on Dental Education......... .......... 178
Series of Clinical Cases......... 384
Soldering Aluminum.......... .. 583
Structural Development..... ... 578
Study of Anatomy............... 519
The Amalgam Question......... 577
The Compatility of Different Filling Materials with Tooth Structure............. 579
The Relations Between the Oral Cavity and the General System ........................ 582
Treatment of Abscess............. 421
Tumors of the Maxillæ......... 365
What is the Best Method for Teaching Children to Care for their Teeth............... 586
What Can We Do to Increase the Attendance in Our Dental Societies?........... 209
What Extent, and When are We Justified in Using Cataphoresis? Is There Danger of Injuring the Dental Pulp or Other Tissues by Its Use?............. 432

### PROCEEDINGS.

Alabama Dental Association.. 319
Annual Address, 1897.......... 458
Discussion on Cataphoresis..... 108
Discolored Structure of Devitalized Teeth—Causes, Method of Treatment and Agents Employed........ .. 20
Michigan Dental Association.. 589
Mississippi Dental Association 325
Mississippi Valley Dental Society......... .................. ...... 140
National Association of Dental Faculties................. 451
Northern Illinois Dental Society............................. 597
Southern Dental Association Abstract of 1896............ 129
Southwestern Michigan Dental Association............... 334
The Evansville Dental Society 596

PAGE.
The Fate of Associations........ 10
Union of the American and Southern Dental Associations......................... ........ 539

### SELECTIONS.

A Bright Lad......... ......... 251
A Plea for a Greater Use of Non-Cohesive Gold......... 397
A Word to the Wise is Sufficient................. .......... 407
Absolute Alcohol as a Disinfectant for Surgical Instruments....................... 191
Administration of Ether to Infants........................ 191
American Dentist Not Wanted in Germany.................. 147
Anecdote Regarding Lord Lister.............................. 303
A New Dressing.................. 598
Are We Longer-Lived than Our Fathers?................ 295
Artificial Camphor.............. 289
Blood Poisoning after Tooth Extraction ...................... 148
Board of Health Statistics Show Favor to Lady Bicylers................ ......... 296
Body and Brain Weariness..... 150
Buffet Cattle Cars ................ 299
Carious Teeth and Tuberculosis ............................ 340
Care of the Teeth........ ........ 252
Cheap Disinfectant................ 101
City Noises versus Health and Longevity......................... 336
Cocaine .............................. 200
Cocaine—Its Uses and Abuse.. 245
Congenstal Teeth ................ 189
Consumption ...................... 71
Commercial Medical Colleges.. 298
Cremation or Burial?............ 302
Death Following the Administration of Nitrous Oxide.. 146
Decision on the Low Bridge Patent .......................... 43
Degree of Anæsthesia Which Should be Induced Prior to Surgical Operations..... 285
Euchinin—A New Preparation of Quinin............... 193
Eucain................................. 192

PAGE.

Ex-Medicos .......................... 151
Exostosis .......................... 226
Extracts from Dr. M. B. Campbell's Annual Report...... 294
Extraction of Teeth and Facial Paralysis .................. 340
Fossil Bacteria .................... 603
Fetid Breath ...................... 295
Formalin .......................... 249
First International Exhibition of Dentistry and Dental Surgery .................... 604
Full, Deep Breathing............ 416
Get Your Pedigree Ready ..... 299
Headaches from Eye Strain.... 604
How to Breathe .................. 183
How to Prescribe ................ 300
How? and Why? ................ 301
How to Write a Medical Paper 195
Humbugging in Medicine...... 403
Hygienic Precepts ................ 296
Hygiene of the First Dentition 297
Incisions Made in the Skin of the Face ...................... 264
Insanity of English People..... 301
Is Consumption Disappearing.. 199
Kaiserling's Method for Preservation of Specimens with their Natural Colors...... 463
Life and Death in the Emotion 196
Longevity .......................... 600
Loss by Typhoid Fever......... 564
Medical Education ............... 399
Medical Superstitions ........... 461
Member of One of the Learned Professions .................... 300
Michigan Dental Association's Annual Meeting ............ 253
Molding of the Superior Maxilla in Adenoid Vegetations ........................... 292
Multum in Parvo ................ 195
New Developments in the Germ Theory of Disease.. 286
New Hæmostatic .................. 304
Nitrous-Oxide Gas with Oxygen .............................. 305
Notes on Tuberculosis—Avenues of Invasion ........... 294
Nothing but Luck ................ 605
Oral Surgery—Theory and Results ............................ 243
Pain and the Surgeon........... 462

PAGE.

Pain and its Therapeusis....... 600
Papain—A Memoir .............. 601
Paracelsus on Doctors. .......... 598
Phlegmon of the Hand......... 198
Preserving Leeches .............. 545
Quinine ............................. 297
Relation of Alcoholic Indulgence to Insanity ............. 147
Remarks on the Treatment of Fractures ....................... 404
Removal of Deposits upon the Teeth ......................... 233
Report of the Commissioner of Education for the Years 1894–95 ...................... 406
Roentgen Rays are Quite Complex .......................... 196
Sickness Rate in Germany..... 187
Silver as an Antiseptic, from Surgical and Therapeutic Viewpoint ................... 400
Some Reminders in Treatment and Diagnosis ............... 188
Spitting Nuisance ................ 185
State Board of Medical Examiners .......................... 599
Struggle for Life.................. 303
Surgical Items .................... 197
The Antiseptic Properties of Saliva ......................... 194
The Art of Not Hearing........ 197
The Chin—Ernest Crutchen... 602
The Influence of Exercise on Growth ....................... 149
The Medicine Habit ............. 401
The Office Hour .................. 402
The Prevention of Consumption .......................... 148
The Spitting Animal ............ 293
The Science of Nutrition........ 304
The Science of the Morning Fast ........................... 290
They Keep Their Hands Clean 298
Then and Now ................... 405
Through the Telephone......... 393
Tobacco and Education......... 289
To Lessen the Number of Dispensaries ....................... 293
124 Years Old .................... 290
United States Supreme Court.. 199
Want a State Prosecuting Agent ........................... 198
When Pay Day Comes .......... 341

PAGE.

COMMENCEMENTS.

Atlanta Dental College......... 354
Baltimore College of Dental Surgery...................... 350
Birmingham Dental College... 340
Boston Dental College.......... 353
Chicago College of Dental Surgery......................... 407
Cincinnati College of Dental Surgery......................... 356
Columbian University—Dental Department............. 354
Dental Department, Southern Medical College............. 343
Detroit College of Medicine... 343
Harvard University—Dental Department.................. 411
Harvard University—Dental Department.................. 351
Kansas City Dental College... 349
Marion Simms College of Medicine—Dental Department 449
Meharry Dental Commencement.............................. 463
Missouri Dental College....... 342
National University—Dental Department.................. 352
New York Dental School...... 352
Northwestern University Dental School..................... 357
Ohio College of Dental Surgery 348
Pennsylvania College of Dental Surgery..................... 410
Philadelphia Dental College.. 408
Tennessee Medical College, Knoxville — Dental Department....................... 411
University of Buffalo........... 342
University of California........ 347
University of Iowa............... 411
University of Michigan......... 356
University of Maryland—Dental Department.............. 346
University of Minnesota........ 344
University of Pennsylvania... 350
University of Tennessee—Dental Department............. 412
Vanderbilt University.......... 355
Western Dental College........ 354
Western Reserve University Dental College.............. 344

PAGE.

EDITORIAL.

American Dental Weekly...... 467
American Medical Association —Dental Section............ 152
Bibliographical...52, 204, 256, ....................310, 468, 566, 606
Catching's Compendium of Practical Dentistry......... 258
Chicago Dental Society......... 50
Cleanliness.......................... 415
Commencements—Dental Colleges.............................. 202
Death of Dr. Frank Abbott and Dr. J. C. Story......... 258
Dental Law in Missouri........ 362
Dental Societies—Directory... 257
DENTISTS IN THE ARMY— Employment of Dentists in the Army..................... 253
Enamel Polish...................... 51
Formaldehyd....................... 364
Frank Abbott, M. D............... 312
Hygienic Resort................... 51
Meeting of the American Dental Association, and Southern Dental Association... 308
Meeting of the National Association of Dental Faculties.............................. 309
Meeting at Old Point Comfort, 1897............................. 464
New College at Indianapolis.. 466
New Work on Surgery.......... 467
Obituary............................ 362
Odontographic Society of Cincinnati........................ 204
Ohio State Dental Society...47, ..............................467, 565
Old Point Comfort................ 307
Programme of the Dental and Oral Section of the American Medical Association.. 259
Questions Submitted for Discussion by Local Societies —Formulated by the Committee Appointed by the American Dental Association....................... 260
Resolutions......................... 362
Southern Indiana Dental Society........................... 50

PAGE.

Tin Foil—H. L. Ambler.......... 606
Toledo Dental Society........... 47
Twelfth International Medical Congress ........................ 361
Vacation......................... .......... 413
Western Kentucky Dental Association ........................ 153
What Is In a Name..... ........ 566
What of Cataphoresis? Is it Practicable? Is it Desirable? .......................... 201

NOTICES.

American Dental Association ........ ......................306, 359
Executive Committee of the American Dental Society of Europe...................... 412
Indiana State Dental Meeting 306
Indiana State Dental Association.......... ...... ............ 564
National Association of Dental Examiners................ 359
National Association of Dental Technics........................ 605
New Jersey State Dental Society .............................. 305
Northern Iowa Dental Society 413

PAGE.

Resolutions of American Academy of Dental Science..... 360
Southern Dental Association.. 358
Southern Dental Association... 605
Southwestern Michigan and Northern Indiana Dental Society........................... 412

MONTHLY DIGEST.

Bacteriology......................... 140
Cataphoresis—Early History.. 32

BIOGRAPHICAL.

In Memoriam—William Newton Morrison, D. D. S...... 101
In Memoriam — Dr. Francis Peabody ........................ 153
In Memoriam—Dr. James A. Swasey........ ................. 155
Dr. E. Magitot...................... 568
Dr. S. B. Brown. The Oldest Practicing Dentist in Fort Wayne . ......................... 206
Dr. Thos. W. Evans.............. 607
Dr. D. R. Jennings................ 609
Dr. W. W. Sheffield.............. 610

### The Chicago Dental Society.

The thirty-third annual meeting of this body will take place on Monday and Tuesday, February 1st and 2d, 1897. Preparation has been made for a large and interesting meeting. This is one of the oldest State Dental Societies of the country; has accomplished much for the profession, and is still one of the most active of this class of organizations. The meeting will convene at 6 o'clock on Monday, February 1st, and will occupy the morning session with clinics. Provision has been made for twenty-five operators. A clinic will also be given in the forenoon of the next day. Papers will be read Monday afternoon and evening and Tuesday afternoon, closing the exercises with a banquet at 6:30 P. M. This will, doubtless, be a very interesting and instructive meeting, and of great value to all who will give attention to what may be done. Headquarters for visitors will be at the Palmer House, where special rates may be obtained. A cordial invitation is extended to all who may find it practicable to attend these meetings. The complete programme will be issued about the middle of January, which will give the location of clinic-rooms, etc.

### THE DENTAL REGISTER,

### A MONTHLY MAGAZINE DEVOTED TO THE INTERESTS OF THE

### DENTAL PROFESSION.

*Terms Reduced to $2.00 Per Year. Single Copies, 25 Cents.*

### EDITED BY PROF. J. TAFT, D.D.S.

—AND—

### Published by SAM'L A. CROCKER & CO., Ohio Dental and Surgical Depot.

The volume commences in January and closes in December.

The REGISTER being issued monthly is always fresh, therefore Indispensable to the practitioner who desires to keep posted up with the new inventions and improvements which are daily developed. Neither expense nor effort will be spared to give value and variety to its contents. Articles will be illustrated with well executed engravings whenever they will add to the interest or assist to explanation.

Its extensive circulation and frequency of issue make it one of the BEST DENTAL ADVERTISING MEDIUMS in the United States.

All subscriptions must begin with January or July.

Local or special notices, five lines or less, will be inserted for $3 each insertion; over five lines, 50 cts. per line.

All advertising bills payable in advance, or at furthest, after the issue of the first number containing the advertisement. All transient advertisements to be paid for in advance.

☞CONTRIBUTIONS SOLICITED.

Exclusively Editorial Communications should be addressed to the Editor.

The latest number of the REGISTER may be ordered with or without other good[illegible] any time, and all letters should be addre[illegible]

